# Comparison of power-free-chop and phaco-chop techniques for moderate nuclei

**DOI:** 10.1186/s12886-020-01455-4

**Published:** 2020-05-01

**Authors:** Lin Yao, Haiqing Bai

**Affiliations:** 1 Qingdao Xinshijie Eye Hospital, 1011st, Dalao Road, Qingdao, 266199 China; 2 Department of Ophthalmology, The Affiliated Hospital of Qingdao University, Qingdao, China

**Keywords:** Power-free-chop, Phaco-chop, Phacoemulsification

## Abstract

**Background:**

To compare the intraoperative and postoperative effects of power-free-chop and phaco-chop techniques for moderate nuclei in phacoemulsification surgery.

**Methods:**

Sixty patients were evaluated in 2 groups. The power-free-chop technique was performed in Group 1 (30 eyes), and the phaco-chop technique was performed in Group 2 (30 eyes). There were no significant differences between these 2 groups. The cumulative dissipated energy (CDE), time to achieve maximum vision, corneal thickness variation, and time to return to the preoperative values were collected. All parameters were statistically compared in these 2 groups by using the chi-square test and the independent-sample *t*-test.

**Results:**

The CDE was 5.53 ± 1.92 J in Group 1 and 7.02 ± 1.77 J in Group 2. After the operation, the mean time to recover to the maximum vision was 2.80 ± 1.42 days in Group 1 and 3.80 ± 1.92 days in Group 2. The mean postoperative corneal thickness increased 36.9 ± 14.74 μm in Group 1 and 46.20 ± 20.67 μm in Group 2. The mean time to return to preoperative pachymetry values was 3.73 ± 1.70 days and 4.83 ± 2.11 days in Group 1 and Group 2, respectively. There were significant differences in these parameters between the groups.

**Conclusions:**

The power-free-chop technique had fewer negative effects on the corneal endothelium, as less ultrasound power was used for moderate nucleus cases. This can accelerate the functional healing process and the return to preoperative physiologic values.

## Background

Complete division of the nucleus is crucial to accomplishing uneventful phacoemulsification. Several chop techniques have been introduced and modified with various advantages and disadvantages [1–6]. The aims of all the techniques are to decrease the ultrasound power used during nucleus emulsification [1].

Among these techniques, phaco-chop is one of the most popular techniques. Although the phaco-chop technique is effective for moderate to hard nuclei, its use with soft nuclei is limited [2, 7]. Therefore, in clinical applications, we invented the power-free-chop technique, which was derived from the modification of the phaco-chop technique. The power-free-chop technique can be used to mechanically cleave soft to moderate nuclei into distinct fragments without ultrasound power participation.

Both power-free-chop and phaco-chop techniques can be used to cleave moderate nuclei. In this study, we compared the efficiency and safety for moderate nucleus cases by using these 2 techniques.

## Methods

This was a retrospective study, which was comprised 60 eyes (60 patients) with cataracts. All of them were performed phacoemulsification using the power-free-chop technique (Group 1, 30 eyes) or the phaco-chop technique (Group 2, 30 eyes). The criteria for these cases were 60–80 years old, 22.0–25.0 mm axial lengths, more than 2000 endothelial cells/mm^2^, anterior chamber depth beyond 2.5 mm, dilated pupil diameter beyond 6 mm, cataract nucleus grade 3 (according to Emery-Little classification [8]) and no other oculopathy.

All surgeries were performed by the same surgeon (L. Y.), who was experienced in these 2 techniques, with a Centurion® phacoemulsification unit (Alcon Laboratories Inc.).

**Fig. 1:**
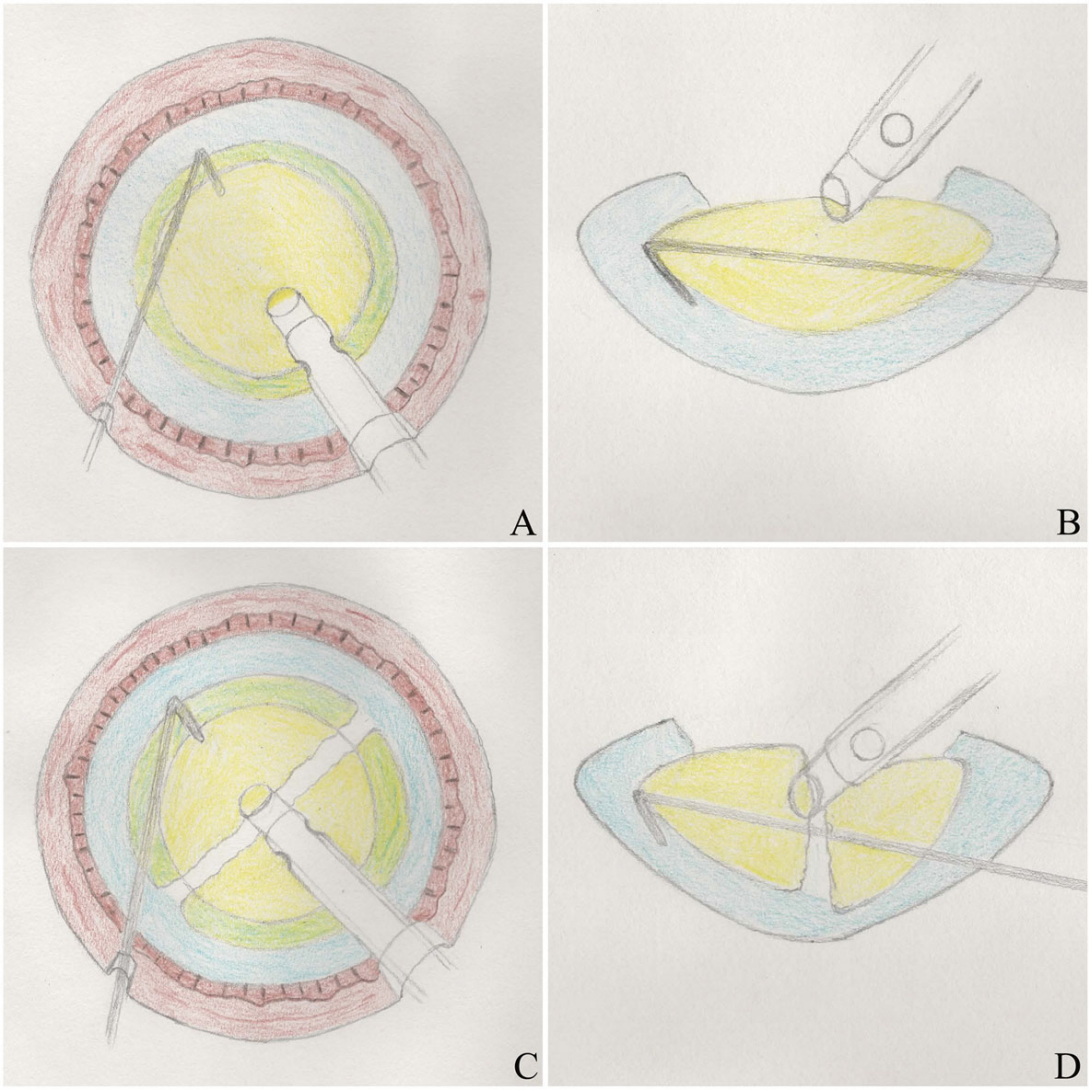
The procedure of power-free-chop technique. The phaco tip was leaned near the geometric centre of the nucleus as deep as possible and the chopper was placed beyond the edge of the nucleus and moved to the phaco tip horizontally (**a**, **b**). The phaco tip was placed in the centre of 2 hemispheres using only irrigation and the chopper was placed beyond the hemisphere (**c**, **d**)

**Fig. 2:**
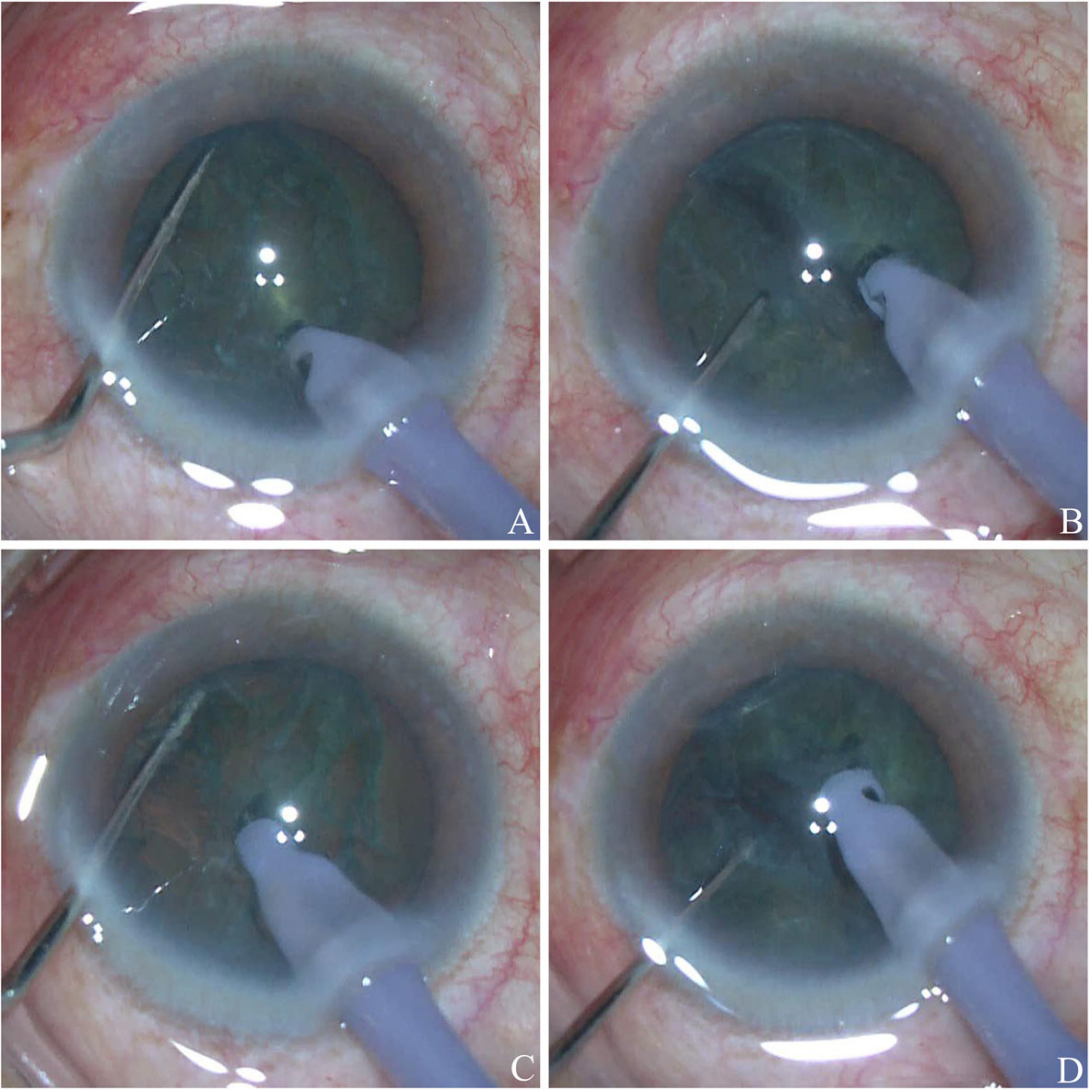
The procedure of power-free-chop technique. The phaco tip was leaned near the geometric centre of the nucleus as deep as possible (**a**). The nucleus was split into 2 hemispheres by the encountered chopper and phaco tip (**b**). The phaco tip was placed in the centre of 2 hemispheres using only irrigation and the chopper was placed beyond the hemisphere (**c**). The chopper moved to the phaco tip horizontally to split the hemisphere into 2 pieces (**d**)

In Group 1 (power-free-chop), a standard 2.75 mm clear corneal incision was made at 11 o’clock, and a side port was created with a stab knife at approximately 4 clock hours away. After continuous curvilinear capsulorhexis was made, hydrodissection and hydrodelineation were performed. The phaco tip was inserted into the anterior chamber, and the superficial cortex was removed. The phaco tip was erected and placed on the nucleus near the capsulorhexis edge at the 11 o’clock position, then it was leaned near the geometric centre of the nucleus as deep as possible using irrigation/aspiration (I/A) gear without ultrasound power (Step 1, foot pedal in position 2, Figs. 1a, b, 2a). The bevel face of the phaco tip was upwards. At this stage, occlusion was not necessary. The chopper was placed beyond the edge of the nucleus and moved to the phaco tip horizontally. Then, the nucleus was split into 2 hemispheres by the encountered chopper and phaco tip (Fig. 2b). Both hemispheres were turned 90 degrees around the horizontal axis using the chopper. The phaco tip was placed in the centre of 2 hemispheres using only irrigation. The chopper was placed beyond the hemisphere, which was opposite the main corneal incision (Step 2, foot pedal in position 1, Figs. 1c, d, 2c). Then, the chopper moved to the phaco tip horizontally to split the hemisphere into 2 pieces (Fig. 2d). Each piece was aspirated and emulsified. Then, the same process was repeated. In the whole process, the position of the chopper was crucial. It must be placed beyond the edge of the nucleus and as deep as possible. I/A was used to clear the remaining cortex. Sodium hyaluronate was injected into the anterior chamber, and a foldable intraocular lens (IOL) was inserted into the capsular bag. The last step was clearing the sodium hyaluronate by I/A, and the corneal incisions were closed by stromal hydration.

**Fig. 3:**
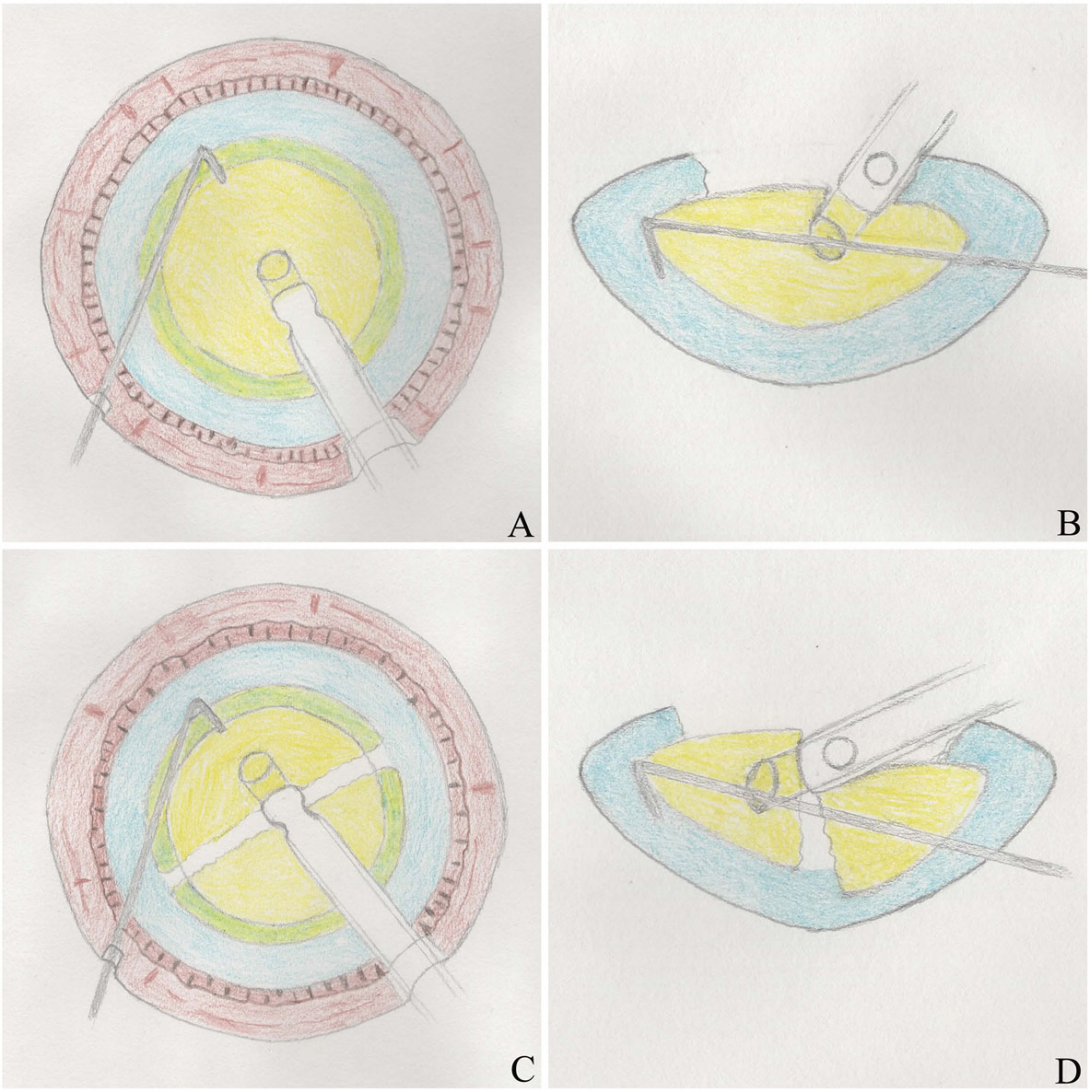
The procedure of phaco-chop technique. The phaco tip was buried in the centre of the nucleus using ultrasound power and the chopper was placed beyond the hemisphere (**a**, **b**). The phaco tip was buried in the centre of 2 hemispheres using ultrasound power and the chopper was placed beyond the hemisphere (**c**, **d**)

Compared with Group 1, there were 2 differences in the operation in Group 2 (phaco-chop). In steps 1 and 2, the phaco tip was buried in the centre of the nucleus with ultrasound power (Fig. 3a-d).

The cumulative dissipated energy (CDE), best corrected visual acuity (BCVA), time to achieve maximum vision, corneal thickness variation, and time to return to the preoperative values (±20 μm according to the preoperative value) were measured postoperatively with OCT (CIRRUS HD-OCT 5000, Carl Zeiss Meditec, Inc.). In the first week, all patients were examined every day, then at an interval of 2 or 3 days in the first month, followed by once every month thereafter.

The chi-square test and the independent-samples *t*-test were used to compare the groups for statistical significance. Data were analysed using SPSS software (version 13.0, International Business Machines Corp.). The level of significance was set to a *P* value of 0.05.

## Results

Thirty patients in each group were evaluated. The characteristics of the patients in these 2 groups are shown in Table 1. There was no statistically significant difference between these 2 groups in any characteristic.

**Table 1:** Patient characteristics

Characteristic	Group 1 (Power-free-chop) (*n* = 30)	Group 2 (Phaco-chop) (n = 30)	*P* Value
Age (y)	70.26 ± 5.60	71.5 ± 5.59	0.396*
Sex (male/female)	12/18	14/16	0.602†
Right eye/left eye	15/15	16/14	0.796†
Preoperative visual acuity (LogMar)	0.56 ± 0.21	0.61 ± 0.18	0.328*
Follow-up period (d)	63.50 ± 16.88	64.43 ± 15.26	0.823*
Preoperative corneal thickness (μm)	534.7 ± 38.37	543.5 ± 37.22	0.371*

Mean ± SD

*Independent-samples *t*-test

†Chi-square test

Between these 2 groups, differences in CDE, time to achieve maximum vision, pachymetry, corneal thickness variation, and time to return to the preoperative values were significant. However, there was no significant difference in postoperative visual acuity between the groups (Table 2). The results showed that compared with the phaco-chop group, the postoperative healing period was shorter in the power-free-chop group.

**Table 2:** Intraoperative and postoperative parameters in the 2 groups

Parameter	Group 1 (Power-free-chop) (n = 30)	Group 2 (Phaco-chop) (n = 30)	*P* Value*
Cumulative dissipated energy (CDE, J)	5.53 ± 1.92	7.02 ± 1.77	0.003
Postoperative visual acuity (LogMar)	0.067 ± 0.076	0.073 ± 0.074	0.732
Time to achieve BCVA (d)	2.80 ± 1.42	3.80 ± 1.92	0.026
Increase in CT (μm)	36.9 ± 14.74	46.20 ± 20.67	0.049
Time to return to preoperative CT (d)	3.73 ± 1.70	4.83 ± 2.11	0.031

Mean ± SD

*BCVA* best corrected visual acuity, *CT* corneal thickness

*Independent-samples *t* test

## Discussion

The chop procedure is the principal step for phacoemulsification. Many methods can be used to crack a nucleus, such as phaco-chop, stop-and-chop, and divide-and-conquer [4, 9]. However, all of them involve occlusion, using high vacuum to stabilize the nucleus and ultrasound power to create the initial groove or fracture.

Among these methods, the phaco-chop technique is the most appropriate method for moderate to hard nuclei [2, 7]. However, it is very difficult to achieve the occlusion and high vacuum required to cleave a soft nucleus. For soft nuclei, even in occlusion, holding is difficult because the phaco tip tends to aspirate the soft nuclear matter. Alternatively, the flipping technique and phaco rolling technique have been described to remove soft nuclei. However, both of them represent a certain risk to the endothelium, particularly in eyes with shallow anterior chambers [10, 11]. Therefore, we invented the power-free-chop technique, which is a modified phaco-chop technique that can be used to mechanically cleave soft nuclei into distinct fragments without occlusion and ultrasound power participation. In clinical applications, we found that the power-free-chop technique is suitable for not only soft nuclei but also moderate nuclei.

Both the power-free-chop and phaco-chop techniques could be used to cleave moderate nuclei. We compared 2 techniques for handling moderate nuclei in our study. The results showed that in the power-free-chop group, CDE was significantly lower. In the postoperative follow-up period, corneal oedema was significantly lower and the healing period was shorter in the power-free-chop group than in the phaco-chop group.

Compared with the phaco-chop technique, occlusion and tight holding are not necessary in the power-free-chop technique. In the chop procedure, the phaco tip just needs to lean against the nucleus as deep as possible in the I/A gear. Therefore, no ultrasound power is wasted in the chop procedure. In contrast, the phaco-chop technique requires ultrasound power to deeply bury the phaco tip into the nucleus. In the chop procedure, the use of the chopper is the same for these 2 techniques. The chopper needs to be placed beyond the edge of the nucleus and move to the phaco tip. Moreover, without ultrasound power involved, the phaco tip will not penetrate the nucleus during the chopping process in the power-free-chop technique. Therefore, this approach can also protect the posterior capsule and avoid the occurrence of posterior capsule rupture.

Another significant benefit of the power-free-chop technique is that this technique does not need to build the occlusion in the nucleus with precise pedal control. This can eliminate the difficulty in the chopping procedure, especially for phaco beginners. Therefore, the power-free-chop technique is easier to learn and control than the phaco-chop and other manual prechop techniques, such as the cystotome-assisted prechop technique [12].

The power-free-chop technique also has disadvantages. It is effective for soft to moderate nuclei (Emery-Little classification grades 1 to 3) only. We have tried to use this technique on hard nuclei, but it did not succeed. If a hard nucleus is encountered, the power-free-chop technique can be easily switched to the phaco-chop technique during the operation.

## Conclusion

In summary, for moderate nuclei, the power-free-chop technique is superior to the phaco-chop technique because it decreased the CDE and accelerated the functional healing process.

## Data Availability

All data generated or analysed during this study are included in this published article.

## Abbreviations

I/A: Irrigation/aspiration
IOL: Intraocular lens
CDE: Cumulative dissipated energy
BCVA: Best corrected visual acuity
